# Assessment of the Awareness of Risk Factors and Current Behavior Among Individuals With Type 2 Diabetes Mellitus in India: A Cross-Sectional Study

**DOI:** 10.7759/cureus.80512

**Published:** 2025-03-13

**Authors:** S S Dariya, Anuj Maheshwari, Vijay Viswanathan, Anil Kumar Virmani, Mohsin Aslam, Alok Modi, Ajoy Kumar Tewari, Ashutosh Chaturvedi, Arun Kumar Kedia, G D Ramchandani, Rajnish Saxena, Jayant K Panda, Ashish Saxena, Akash N Singh, Bijay Patni, Ashish S Dengra, Dhruvi Hasnani, Vipul Chavda, Kannan Natarajan, Anubha Varma, Aravinda Jagadeesha, Dinesh Agarwal, Nagendra Kumar Singh, L Sreenivasamurthy, Bharat Saboo, Anil Samaria, Sandeep Suri, Sajid Ansari

**Affiliations:** 1 Internal Medicine, National Institute of Medical Sciences and Research, Jaipur, IND; 2 Internal Medicine, Hind Institute of Medical Sciences, Lucknow, IND; 3 Diabetes and Endocrinology, Prof. M. Viswanathan Hospital for Diabetes, Chennai, IND; 4 Internal Medicine, Dr. Anil Kumar Virmani Clinic, Jamshedpur, IND; 5 Internal Medicine, Asian Institute of Gastroenterology, Hyderabad, IND; 6 Internal Medicine, Kevalya Hospital, Thane, IND; 7 Internal Medicine, Jai Clinic & Diabetes Care Center, Lucknow, IND; 8 Internal Medicine, Mahatma Gandhi Hospital, Jaipur, IND; 9 Internal Medicine, Lifeworth Hospital, Raipur, IND; 10 Internal Medicine, Ramchandani Diabetes Care and Research Centre, Kota, IND; 11 Diabetes and Endocrinology, Saxena Diabetes Care Centre, Ajmer, IND; 12 Internal Medicine, Srirama Chandra Bhanja Medical College and Hospital, Cuttack, IND; 13 Medicine and Metabolic Disease, Clinical Cardiology, Diabetes and Heart Centre, Ludhiana, IND; 14 Internal Medicine, Manjalpur Hospital, Vadodara, IND; 15 Internal Medicine, Diabetes Wellness Care, Kolkata, IND; 16 Diabetology, Mahi Diabetes Thyroid Care and Research Centre, Jabalpur, IND; 17 Diabetology, Rudraksha Institute of Medical Sciences, Ahmedabad, IND; 18 Internal Medicine, Rudraksha Institute of Medical Sciences, Ahmedabad, IND; 19 Internal Medicine, Chandra Chest and Diabetes Care Centre, Chennai, IND; 20 Internal Medicine, Moti Lal Nehru Medical College, Prayagraj, IND; 21 Internal Medicine, Dr. Aravind’s Diabetes Centre, Bengaluru, IND; 22 Internal Medicine, Marwari Hospital and Research Centre, Gawahati, IND; 23 Internal Medicine, Diabetes and Heart Research Centre, Dhanbad, IND; 24 Internal Medicine, Lifecare Hospital and Research Centre, Bengaluru, IND; 25 Diabetology, Prayas Diabetes Center, Indore, IND; 26 Internal Medicine, Jawaharlal Nehru Medical College, Ajmer, IND; 27 Internal Medicine, Advance Diabetes and Critical Care, Hisar, IND; 28 Internal Medicine, SS Heart Care Centre, Lucknow, IND

**Keywords:** awareness, diabetic complications, knowledge, risk factors, type 2 diabetes mellitus

## Abstract

Background

The burden of type 2 diabetes mellitus (T2D) is compounded by serious complications, including cardiovascular and microvascular diseases, with significant healthcare costs associated with these complications. Engaging in self-care practices can enhance glycemic control and empower individuals to make informed health decisions. The present study aims to assess the existing levels of awareness and understanding of diabetes, focusing on the perception of its risk factors and associated complications.

Methodology

This prospective, cross-sectional study involved 2,468 individuals with T2DM recruited from 26 sites across India. Data underwent aggregate-level analysis using Python and were assessed for statistical significance using the chi-square test. Continuous variables (means and standard deviations) were analyzed, and differences among groups were evaluated using analysis of variance. To assess the understanding of diabetes, including its symptoms and complications, all knowledge-related questions were aggregated into a composite score. Participants were categorized into three groups (high, medium, and low) based on their knowledge levels. The participant’s variables across five dimensions, namely, diabetes causes and symptoms, medication usage, diet and lifestyle, hypoglycemia, and complications from diabetes, were assessed. The highest achievable score was 26, while the lowest was 0. Based on the knowledge score, individuals were classified into the following three groups: high for participants who scored 80% or more correct answers (score >20); medium for those scoring between 40% and 80% (score between 11 and 20); and low for participants with a score between 0 and 10, indicating fewer than 40% correct responses.

Results

The average participant age was 54.1 years, with a male-to-female ratio of 60:40. The average hemoglobin A1c level was 8.2%, with 629 (28%) maintaining levels within the target range (≤7%), and 497 (22%) having levels above 9%. Overall, 655 (26.50%) individuals were classified as high knowledge, 1,179 (47.77%) as medium knowledge, and 634 (25.68%) as low knowledge. Scores were 22.4 (1.2) for high knowledge, 15.9 (2.9) for medium, and 6.2 (3.0) for low. Diabetic complications were significantly higher in the high-knowledge group. Adherence to diabetes medication was higher in the high (509 (78.5%)) and medium (917 (81.6%)) knowledge groups (p < 0.001) compared to the low (363 (60.3%)) knowledge group. Participants with high and medium knowledge levels demonstrated significantly greater ownership and use of glucometers compared to those with lower knowledge levels.

Conclusions

Individuals with T2DM who experience complications tend to have a greater understanding of diabetes and its risk factors than those without complications. This suggests that facing health issues motivates individuals to seek information, improving their knowledge and influencing better medication adherence and lifestyle choices. The findings of this study, coupled with existing data, underscore the urgent need for innovative approaches to engage and motivate individuals through targeted educational initiatives, comprehensive counseling, regular monitoring, and strategies to improve adherence to recommended diabetes management practices.

## Introduction

The prevalence of non-communicable diseases is on the rise, with India as the primary contributor in South Asia [1]. According to the ICMR-INDIAB-17 study, 101.3 million people had diabetes and 136 million people had pre-diabetes in India (overall weighted prevalence of 11.4% and 15.3%, respectively) [2].

Indians appear to have a higher susceptibility to diabetes, primarily due to an elevated degree of insulin resistance and a stronger genetic predisposition [3]. Contributing to this trend are factors such as obesity (central obesity and increased visceral fat), along with the consumption of high-calorie, high-fat, and high-sugar diets [3,4]. The burden of type 2 diabetes mellitus (T2DM) increases due to its association with macrovascular (cardiovascular, cerebrovascular, and peripheral artery diseases) and microvascular (diabetic retinopathy, nephropathy, and neuropathy) complications [5]. In a systematic review, the prevalence rates of chronic diabetes complications were retinopathy ranging from 4.8% to 21.7%, nephropathy from 0.9% to 62.3%, and neuropathy from 10.5% to 44.9% [6]. Studies indicate that illness costs are 1.4 times higher for individuals experiencing complications [7,8].

These statistics highlight the critical importance of quality diabetes management in India to alleviate the healthcare and economic burden. Self-management plays a vital role in improving outcomes for individuals with T2DM. By actively engaging in self-care practices, such as monitoring blood glucose levels, adhering to medication regimens, and maintaining a healthy lifestyle, individuals can achieve better glycemic control and reduce the risk of complications. Furthermore, self-management fosters a sense of empowerment and confidence, enabling individuals to make informed decisions about their health and collaborate effectively with healthcare providers for optimal care.

Studies on knowledge, attitude, and practice have yielded substantial evidence emphasizing the imperative requirement for heightened awareness among the general population and individuals with diabetes, specifically concerning prevention, managing risk factors, and disease control [9-11]. Effective education and awareness initiatives can transform the general population’s attitudes toward diabetes [12].

We searched PubMed, Google Scholar, and Scopus for studies conducted among Indians on this topic. The focus was to assess studies conducted recently (the last 10 years). There are several single-center and regional studies conducted over time. Most studies concluded that appropriate knowledge and attitude regarding diabetes mellitus to some degree exists, but there are important lacunae and practices that are often found wanting. No large-scale, pan-India studies have attempted to map the level of knowledge, attitude, and practice among people with T2DM. Hence, this study aims to evaluate the current levels of awareness, perceptions of risk factors, and complications of diabetes while categorizing them based on their awareness and practices.

## Materials and methods

Study design and population

This was a prospective, cross-sectional study among individuals with T2DM in urban India. Before the main study, a pilot study with five doctors was conducted, and the questionnaire was validated. The study was conducted from April 14, 2023, to July 15, 2023. An electronic case report form (eCRF) in the form of a questionnaire was used to collect data for assessing awareness and attitudes toward T2DM management.

Inclusion and exclusion criteria

The study included adults aged 18 years or older who were diagnosed with T2DM and provided consent. Exclusion criteria comprised individuals younger than 18 years, those diagnosed with T2DM for fewer than 12 months at the time of the study, and individuals diagnosed with other types of diabetes, including type 1 diabetes mellitus, gestational diabetes mellitus, or maturity-onset diabetes of the young.

Study sampling

The recruitment was conducted during routine outpatient consultations. Each site had a designated study assistant responsible for conducting subject interviews. When an individual with T2DM visited the site for a consultation, the site’s principal investigator (PI) assessed their eligibility and asked whether they would be willing to participate in the study. If the subject agreed, they were referred to the study assistant, who provided detailed study information and written informed consent. If the subject declined, the process was terminated, and no data were collected. Once consent was obtained, the interview was conducted in person, and responses were recorded in an eCRF. As soon as the study assistant was free, the PI repeated the process with the next eligible subject. On average, each site conducted 8-10 complete interviews per day. The first 100 eligible consecutive people with T2DM who visited the study site for routine care during the recruitment period and provided informed consent were included. The recruitment phase identified 2,752 subjects, with 2,468 meeting all the criteria for inclusion in the final analysis. Given the study’s observational nature, randomization was not employed. As the study focused on a predefined individual cohort with diabetes, calculations for sample size were deemed unnecessary.

Data collection instrument and variables

Data were collected via in-person interviews with eligible participants during their outpatient department visits at study sites. All interviews were conducted by a trained interviewer/site PI using a structured questionnaire.

The questionnaire was structured into two distinct sections. Section A comprised clinical data typically obtained during routine consultations: demographics, vital signs, diagnosis, complications, medication class, and laboratory assessments. Responses were collected in Section B, focusing on their current behaviors, perceptions, and attitudes regarding various risk factors, alternative medicine, and mobile application-based programs. The questionnaire was developed by integrating items from four widely used and validated scales that assess diabetes knowledge, self-care behaviors, and risk factor awareness. These include the Diabetes Knowledge Questionnaire, the Summary of Diabetes Self-Care Activities measure, the Michigan Diabetes Knowledge Test, and the Diabetes Risk Test. Each of these scales was reviewed by a panel of experts. Relevant questions from these scales were selected that aligned with the study objectives, particularly those addressing diabetes symptoms, risk factors, medication adherence, lifestyle modifications, and complications.

Informed consent and ethics approval

The study was approved by Tanvir Hospital Institutional Ethics Committee for Biomedical and Health Research (approval number: #EC/NEW/INST/2022/TE/0154) on March 31, 2023, Ref 03/2023. Informed consent was obtained in writing and clarified verbally, with study details explained. Physicians maintained confidentiality as per their agreement, and the individuals’ data were anonymized using unique physician identifiers. Data usage was restricted solely to this study, with any further use contingent on additional written permission to protect privacy and uphold ethical standards.

Statistical analysis

The collected data underwent aggregate-level analysis using Python software (version no. 3.86). Categorical data were presented as frequencies and proportions, with statistical significance assessed using the chi-square test. Statistical analyses and visualizations were performed using Microsoft Office. Significance was determined using standard hypothesis testing with a threshold of a p-value less than 0.05. All tests adhered to methodological guidelines to ensure the validity and reliability of the study’s conclusions.

Data presentation and analysis

This study presented data using tables for better interpretation. Continuous variables were described by means and standard deviations. Analysis of variance (ANOVA) was utilized to compare mean differences among groups.

Creation of a composite score and reasoning 

To assess individuals’ responses to diabetes, its symptoms, and complications, all knowledge questions were combined into a single score. As this study’s main objective was the assessment of risk, the weightage was higher for the same. The distribution of weightage across different categories was as follows: diabetes causes and symptoms = 6; medication continuation = 1; diet and lifestyle = 5; hypoglycemia = 1; and complications from diabetes = 13 (refer to the Appendix for the questionnaire).

Each correct response held equal weight in this score. The highest achievable score was 26, while the lowest was 0. Based on this knowledge score, individuals were classified into the following three groups as follows (all scores were of a maximum possible score of 26) (Table 1): high for participants scoring 80% or more correct answers (score >20); medium for those scoring between 40% and 80% (score between 11 and 20); and low for participants with a score between 0 and 10, indicating less than 40% correct responses.

**Table 1:** The scoring system adopted in this study.

Weight	Question number	About diabetes mellitus	Overall, N (%)
1	Diabetes is a condition in which the body contains		
	1	A higher level of sugar in the blood than normal	1,625 (66.2)
	-	A lower level of sugar in the blood than normal	157 (6.4)
	-	Either a higher or a lower level of sugar in the blood than normal	326 (13.3)
	-	I don’t know	345 (14.1)
1	The major cause of diabetes is		
	-	An increased availability of insulin in the body	285 (11.67)
	2	A decreased availability of insulin in the body	1,294 (53.2)
	-	I don’t know	855 (35.1)
-	The symptom(s) of diabetes is/are		
1	3	Increased frequency of urination	1,970 (79.8)
1	4	Increased thirst and hunger	1,848 (74.9)
1	5	Increased tiredness	1,779 (72.1)
1	6	Slow healing of wounds	1,511 (61.2)
-	-	I don’t know	237 (9.6)
-	The lifestyle modification(s) required for individuals with diabetes		
1	7	Weight reduction	1,772 (71.8)
1	8	Balanced diet	1,936 (78.4)
1	9	At least 30 minutes of physical exercise daily	1,826 (74..0)
1	10	Stop smoking	1,439 (58.3)
1	11	Stop alcohol intake	1,405 (56.9)
-	-	I don’t know	215 (8.7)
1	Upon control of diabetes, the medicines		
	-	Can be stopped immediately	85 (3.6)
	-	Can be stopped after a month	154 (6.5)
	12	Should continue with medications	1,344 (56.5)
	-	Not sure	794 (33.4)
-	Which according to you are complications due to uncontrolled diabetes?		
1	13	Eye problems/Blindness	1,730 (70.1)
1	14	Amputation	671 (27.2)
1	15	Kidney disease	1,858 (75.3)
1	16	Heart disease	1,682 (68.2)
1	17	Neuropathy	1,095 (44.4)
1	18	Foot ulcers	1,585 (64.2)
1	19	Shortness of breath	337 (13.7)
1	20	Chest pain while walking	304 (12.3)
1	21	Sexual problems	329 (13.3)
-	-	I don’t know	419 (17.0)
-	In an individual with diabetes, high blood pressure can increase or worsen		
1	22	The risk of heart attack	1,800 (72.9)
1	23	The risk of stroke	1,552 (62.9)
1	24	The risk of eye problems	1,518 (61.5)
1	25	The risk of kidney problems	1,504 (60.9)
-	-	I don’t know	345 (14.0)
-	Are you aware of blood sugar levels falling below normal when you take medicine?		
1	26	Yes	830 (35.1)
-		No	1,538 (64.9)

There are no established benchmarks or validated composite scoring methods for this classification. However, after a thorough review of the results, the study group determined that the defined groups exhibit significant differences and show a strong correlation with behavioral patterns, making them clinically relevant for interpretation and action planning. The study group strongly recommends a follow-up study to further refine the tool and evaluate its generalizability across different cohorts, including variations by region, gender, and age.

## Results

Patient profile

A total of 2,468 people with T2DM across 26 centers in India participated in this study. The average age was 54.1 (±12.2) years, with 864 (35%) in the <50-year age group. Overall, 1,482 (60%) (95% confidence interval (CI) = 58.1-61.9) of the sample were males, and 986 (40%) (95% CI = 38.1-41.9) were females. The average body mass index (BMI) was 27.0 (±9.0) kg/m², with 1,529 (61.9%) in the >25 kg/m^2^ BMI group. Overall, 1,068 (43.2%) had high blood pressure (>140/90 mmHg) during inclusion in the study. The average duration of T2DM was 7.2 (±6.2) years. Moreover, 1,964 (79.6%) were on metformin as monotherapy or combination therapy, 1,086 (44%) were on sulphonylureas, 1,003 (40.6%) were on dipeptidyl-peptidase 4 inhibitors, 682 (27.6%) were on sodium-glucose cotransporter 2 inhibitors, and 392 (15.9%) were on insulin. At the time of inclusion in this study, the mean hemoglobin A1c (HbA1c) level was 8.2 (±1.9). Only 629 (27.9%) of the participants had HbA1c levels within the target range (≤7%), while 497 (22.1%) had HbA1c levels surpassing 9%. Among these complications, neuropathy had the highest prevalence at 878 (37.3%), followed by diabetic foot at 484 (20.6%), retinopathy at 458 (19.5%), and nephropathy at 368 (15.6%). Of the participants, 1,731 (76.7%) had at least one known comorbidity. Hypertension was the most prevalent comorbid condition at 1,337 (57%) (Table 2).

**Table 2:** Study sample profile. SBP = systolic blood pressure; DBP = diastolic blood pressure

Characteristics	N (%)
Age	N = 2,468
<50 years, n (%)	864 (35.0)
50–60 years, n (%)	841 (34.1)
>60 years, n (%)	763 (30.9)
Average (SD)	54.1 (12.2)
Gender	N = 2,468
Male, n (%)	1,482 (60)
Female, n (%)	986 (40)
Body mass index	N = 2,354
Underweight (<18.5 kg/m²)	52 (2.2)
Normal weight (18.5–22.99 kg/m²)	368 (15.6)
Overweight (23.0–24.9 kg/m²)	405 (17.2)
Pre-obesity (25.0–29.9 kg/m²)	1,069 (45.4)
Obesity (≥30 kg/m²)	460 (19.5)
Average (SD)	27.0 (9.0)
Blood pressure	N = 2,431
Optimal blood pressure (SBP: <130/DBP: <85)	790 (32.5)
High normal blood pressure (SBP: 130–139/DBP: 85–89)	573 (23.6)
Grade 1 hypertension (SBP: 140-159/DBP: 90–99)	855 (35.2)
Grade 2 hypertension (SBP: ≥160/DBP: ≥100)	213 (8.8)
Current medication	N = 2,468
Metformin	1,964 (79.6)
Sulfonylureas	1,086 (44)
Dipeptidyl peptidase 4 inhibitors	1,003 (40.6)
Sodium-glucose cotransporter-2 inhibitors	682 (27.6)
Semaglutide	128 (5.2)
Insulin	392 (15.9)
Others	84 (3.4)
Duration of diabetes	N = 2,466
<5 years	944 (38.3)
5–10 years	1,024 (41.5)
>10 years	498 (20.2)
HbA1c	N = 2,251
≤7%	629 (27.9)
7–7.9%	715 (31.8)
8–8.9%	410 (18.2)
>9%	497 (22.1)
Average (SD)	8.2 (1.9)

Knowledge level grouping

The average knowledge score among participants was 15.1 (±6.4) out of a maximum of 26. Based on their composite scores, participants were classified into three groups. The high-knowledge group, with a composite score of 22.4 (±1.2) and a cutoff score >20, comprised 655 (26.50%) subjects. The medium-knowledge group, scoring between 11 and 20 with a composite score of 15.9 (±2.9), accounted for 1,179 (47.77%) participants. Lastly, the low-knowledge group, with a composite score of 6.2 (±3.0) and a cutoff score ≤10, included 634 (25.68%) subjects.

Comparison based on knowledge groups

Patient Characteristics 

Participants with high knowledge levels (mean age = 51.7 (12.4) years) were younger than those with medium knowledge levels (mean age = 55.4 (11.3) years) and low knowledge levels (mean age = 54 (13.3) years) (p < 0.001). Women had lower knowledge levels than men (p < 0.001). No significant differences (p > 0.05) in the knowledge group were noted by duration of diabetes (p = 0.20), BMI (p = 0.98), and current HbA1c levels (p = 0.058). Diabetic complications were significantly higher in the higher knowledge group compared to the medium and lower knowledge groups (p < 0.001) (neuropathy: 47% vs. 41.1% vs. 21.3%, p < 0.001; diabetic foot: 26.3% vs. 20.6% vs. 15.1%, p < 0.001; retinopathy: 28.9% vs. 17.8% vs. 13.6%, p < 0.001; nephropathy: 22.1% vs. 16% vs. 9%, p < 0.001). Similarly, the higher knowledge group had a higher prevalence of comorbid conditions than other groups (p < 0.001) (Table 3).

**Table 3:** Comparison of patient characteristics based on knowledge level groups. ANOVA = analysis of variance; CI = confidence interval

Characteristics	Knowledge level grouping (score)					
	All	Low (≤10)	Medium (11–20)	High (>20)	P-value	Statistical test
N	2,468	634	1179	655	-	-
Age (years)						
<50 years, n (%)	864 (35.0)	223 (35.2)	350 (29.7)	291 (44.4)	<0.001	Chi-square test
50–60 years, n (%)	841 (34.1)	205 (32.3)	429 (36.4)	207 (31.6)		
>60 years, n (%)	763 (30.9)	206 (32.5)	400 (33.9)	157 (24.0)		
Mean (SD)	54.1 (12.2)	54.0 (13.3)	55.4 (11.3)	51.7 (12.4)	<0.001	ANOVA
Gender						
Male, % (95% CI)	60 (58.1-61.9)	50.3 (46.4-54.2)	62.9 (60.1-65.7)	64.3 (60.6-68.0)	<0.001	Chi-square test
Female, % (95% CI)	40 (38.1-41.9)	49.7 (45.8-53.6)	37.1 (34.3-39.9)	35.7 (32.0-39.4)		
Body mass index						
Mean (SD)	27.0 (9.0)	27.0 (5.0)	26.9 (6.8)	26.9 (14.0)	0.977	ANOVA
HbA1c						
Mean (SD)	8.2 (1.9)	8.4 (2.1)	8.1 (1.7)	8.2 (1.9)	0.058	ANOVA
Duration of diabetes mellitus						
<5 years	944 (38.3)	266 (42.0)	432 (36.7)	246 (37.6)	0.2	Chi-square test
5–10 years	1,024 (41.5)	240 (37.9)	504 (42.8)	280 (42.7)		
>10 years	498 (20.2)	128 (20.2)	241 (20.5)	129 (19.7)		
Mean (SD)	7.2 (6.1)	6.9 (6.2)	7.3 (5.9)	7.3 (6.2)	0.428	ANOVA
Diabetes complications						
Neuropathy, % (95% CI)	37.3 (35.3-39.3)	21.3 (18.1-24.5)	41.1 (38.2-44.0)	47.0 (43.0-51.0)	<0.001	Chi-square test
Diabetic foot, % (95% CI)	20.6 (19.0-22.2)	15.1 (12.3-17.9)	20.6 (18.3-22.9)	26.3 (22.7-29.9)		
Retinopathy, % (95% CI)	19.5 (17.9-21.1)	13.6 (10.9-16.3)	17.8 (15.6-20.0)	28.9 (25.2-32.6)		
Nephropathy, % (95% CI)	37.3 (35.3-39.3)	21.3 (18.1-24.5)	41.1 (38.2-44.0)	47 (43.0-51.0)		
Coronary heart disease, % (95% CI)	8.5 (7.4-9.6)	12.3 (9.7-14.9)	8.2 (6.6-9.8)	5.1 (3.3-6.3)		
With any complication, % (95% CI)	59.3 (57.4-61.2)	48.9 (45.0-52.8)	60.6 (57.8-63.4)	67.2 (63.6-70.8)		
Comorbidity						
Hypertension, % (95% CI)	57 (55.0-59.0)	46.2 (42.3-50.1)	60.4 (57.5-63.3)	61.9 (58.0-65.8)	<0.001	Chi-square test
Dyslipidemia, % (95% CI)	28.7 (26.9-30.5)	17.5 (14.5-20.5)	31.3 (28.6-34.0)	35.4 (31.6-39.2)		
Hypothyroidism, % (95% CI)	11.1 (9.8-12.4)	7.9 (5.8-10.0)	11.9 (10.0-13.8)	12.9 (10.1-15.7)		
Hyperthyroidism, % (95% CI)	2 (1.4-2.6)	1.3 (0.4-2.2)	1.5 (0.8-2.2)	3.5 (2.0-5.0)		
With any Comorbidity, % (95% CI)	75 (73.3-76.7)	66.7 (63.0-70.4)	77.2 (74.8-79.6)	79.2 (76.1-82.3)		
Counseling about complications						
Yes, recently, % (95% CI)	40.1 (38.1-42.1)	30.6 (26.9-34.3)	38.9 (36.1-41.7)	51 (47.1-54.9)	<0.001	Chi-square test
Yes, sometime back, % (95% CI)	49.3 (47.3-51.3)	49.1 (45.1-53.1)	50.7 (47.8-53.6)	47.1 (43.2-51.0)		
No, % (95% CI)	10.6 (9.4-11.8)	20.4 (17.2-23.6)	10.4 (8.6-12.2)	1.9 (0.8-3.0)		

Behavioral Differences

Medication adherence: High (509 (78.5%)) and medium-knowledge (917 (81.6%)) participants had higher seven-day adherence to diabetes medication compared to low-knowledge (363 (60.3%)) participants (p < 0.001).

Exercise: Overall, 222 (39.8%) low, 217 (20.2%) medium, and 50 (8.1%) (p<0.001) high-knowledge participants did no exercise in the past seven days. On average, participants with high knowledge exercised for more than 30 minutes on 4.7 (2.3) days, while those with low knowledge exercised on 2.8 (2.7) days.

Diet counseling and eating habits: Overall, 171 (28.2%) participants with low knowledge had received no diet counseling in the last year. This was significantly lower (p < 0.001) among medium-knowledge (120 (10.5%)) and high-knowledge participants (50 (7.8%)). Among participants with high knowledge levels, 383 (59.6%) received counseling from dietitians, and 183 (28.5%) from doctors. Overall, 2,081 (87.2%) participants had regular breakfast. This was higher in participants with high knowledge compared to those with low knowledge (598 (92.9%) vs. 493 (81.8%), p < 0.001).

Self-Assessment Habit

Ownership and usage of a glucometer: Compared to low-knowledge participants (221 (36.4%)), high (423 (65.2%)) and medium-knowledge (772 (67.7%)) participants had higher ownership of a glucometer (p < 0.001) and usage for self-assessment of blood glucose (66 (12.0%), 158 (15.7%), and 259 (44.2%), respectively, p < 0.001).

Diabetic foot: High and medium-knowledge participants had self-examined their feet more in the last month than low-knowledge participants (491 (75.5%), 607 (53.4%), and 273 (45.0%), respectively, p < 0.001). Significantly higher (196 (32.3%), p < 0.001) participants with low knowledge had never checked their feet for diabetic foot complications (Table 4).

**Table 4:** Behavior and sleep. ANOVA = analysis of variance; BP = blood pressure

Characteristics	Knowledge level grouping (score)					
	All	Low (≤10)	Medium (11–20)	High (>20)	P-value	Statistical test
N	2,468	634 (25.68%)	1,179 (47.77%)	655 (26.50%)		
In the last 7 days, on how many days did you take your diabetes medications as prescribed by the doctor, n (%)						
Did not take medications	100 (4.2)	38 (6.3)	46 (4.1)	16 (2.5)	<0.001	Chi-square
Up to 4 days	201 (8.5)	110 (18.2)	65 (5.7)	26 (4.1)		
5 days	143 (6.0)	52 (8.6)	43 (3.8)	48 (7.4)		
6 days	141 (5.9)	39 (6.5)	53 (4.7)	49 (7.6)		
7 days	1,789 (75.4)	363 (60.3)	917 (81.6)	509 (78.5)		
From whom have you received diet counseling in the last 1 year? n (%)						
Doctor	962 (40.2)	246 (40.5)	533 (46.7)	183 (28.5)	<0.001	Chi-square
Dietitian	978 (40.9)	155 (25.5)	440 (38.5)	383 (59.6)		
Family member/Friend	76 (3.2)	27 (4.4)	34 (3.0)	15 (2.3)		
Online	27 (1.1)	6 (1.0)	12 (1.1)	9 (1.4)		
No one	341 (14.3)	171 (28.2)	120 (10.5)	50 (7.8)		
Do you have breakfast regularly? n (%)						
Yes	2,081 (87.2)	493 (81.8)	990 (86.9)	598 (92.9)	<0.001	Chi-square
No	305 (12.8)	110 (18.2)	149 (13.1)	46 (7.1)		
How would you classify your appetite, n (%)						
Normal	1,893 (79.7)	446 (74.2)	929 (81.8)	518 (81.2)	<0.001	Chi-square
Poor	315 (13.3)	112 (18.6)	147 (12.9)	56 (8.8)		
Excessive	167 (7.0)	43 (7.2)	60 (5.3)	64 (10.0)		
On how many of the last seven days did you participate in at least 30 minutes of physical activity? (total minutes of continuous activity, including walking)						
Mean days (SD)	3.8 (2.7)	2.8 (2.7)	3.8 (2.7)	4.7 (2.3)	<0.001	ANOVA
How many hours of sleep do you typically get in a day?						
Mean hours (SD)	7.0 (1.4)	7.1 (1.5)	7.0 (1.5)	7.1 (1.4)	0.105	ANOVA
Do you have a glucose meter and use the same to regularly monitor blood glucose at home/place of work, n (%)						
No, do not have a glucose meter	981 (40.9)	386 (63.6)	369 (32.3)	226 (34.8)	<0.001	Chi-square
Have a glucose meter, but rarely use it	443 (18.5)	83 (13.7)	227 (19.9)	133 (20.5)		
Have a glucose meter, but use it sometimes when needed	693 (28.9)	102 (16.8)	369 (32.3)	222 (34.2)		
Have a glucose meter, and use it regularly, i.e., at least a few times every week	280 (11.7)	36 (5.9)	176 (15.4)	68 (10.5)		
Do you have a BP monitoring device and use the same to regularly monitor BP at home/place of work, n (%)						
No, do not have a BP monitoring device	1,364 (57.1)	460 (75.9)	537 (47.3)	367 (56.7)	<0.001	Chi-square
Have a BP monitoring device but rarely use it	342 (14.3)	57 (9.4)	172 (15.2)	113 (17.5)		
Have a BP monitoring device, but use it sometimes when needed	505 (21.1)	71 (11.7)	297 (26.2)	137 (21.2)		
Have a BP monitoring device and use it regularly, i.e., at least a few times every week	177 (7.4)	18 (3.0)	129 (11.4)	30 (4.6)		
When did you check your feet last? n (%)						
Within the last 7 days	575 (24.0)	157 (25.9)	256 (22.5)	162 (24.9)	<0.001	Chi-square
Within the last 2 weeks	451 (18.8)	66 (10.9)	200 (17.6)	185 (28.5)		
Within the last 6 months	161 (6.7)	30 (5.0)	103 (9.1)	28 (4.3)		
Greater than 6 months	225 (9.4)	55 (9.1)	130 (11.4)	40 (6.2)		
In the last 1 month	345 (14.4)	50 (8.3)	151 (13.3)	144 (22.2)		
In the last 2 months	223 (9.3)	52 (8.6)	118 (10.4)	53 (8.2)		
None	413 (17.3)	196 (32.3)	179 (15.7)	38 (5.8)		

## Discussion

Early detection and treatment of T2DM complications are crucial for preventing progression and enhancing overall quality of life. With the rising prevalence and a very large uncontrolled T2DM population, this study evaluated the awareness of T2DM risk factors and complications. It also examined the current behaviors of individuals with T2DM to gain insights into their adherence to recommended lifestyle modifications and medications.

In the present study, 655 (26.50%) had high knowledge, 1,179 (47.77%) had medium knowledge, and 634 (25.68%) had low knowledge. Our results show an improvement compared to the study conducted among 1,051 adult individuals by Sękowski et al. (2022), where only 17.3% had good knowledge, 46.3% had moderate knowledge, and 36.3% had poor knowledge [13]. Similarly, Chavan et al. (2015) in a single-center study in a rural health center (n = 307) in India reported even lower awareness levels, with only 9.4% of participants having good knowledge, 71.3% having moderate knowledge, and 19.2% having poor knowledge [14]. Mumu et al. (2014), who assessed knowledge among tertiary-care hospital attendees in Bangladesh (n = 400), found that 19% had poor knowledge, 68% had average knowledge, and 13% had good knowledge [15]. The more recent study by Theivasigamani et al. (2023) in rural Tamil Nadu (n = 974), India, reported better results than our study, with 40.9% having good knowledge, 46.3% having average knowledge, and only 7.8% having poor knowledge, with no significant difference in knowledge between males and females [16]. Older studies, such as by Chavan et al. [14] and Mumu et al. [15], reported lower knowledge levels, reflecting the awareness levels at that time. More recent studies, such as Sękowski et al. [13], Theivasigamani et al. [16], and the present study, indicate a trend of increasing knowledge, likely due to improved health education and awareness programs. In the present study, males had a higher level of knowledge compared to females (p < 0.001), which is supported by Deepa et al. [17], Mathur et al. [18], Chavan et al. [14], and Chaudhary et al. [19] and contradicted by Lemes Dos Santos et al. [20], Li et al. [21], and Salleh et al. [22]. In India, knowledge levels tend to be lower among females, possibly due to a greater emphasis on prioritizing family health over their own. A systematic review of 21 articles reported that women encounter various barriers in accessing T2DM care, including personal, sociocultural, health system, economic, psychological, and geographical factors [23].

In the present study, 83% were aware of complications due to diabetes, with the highest being kidney disease (75.3%). According to Deepa et al. [17], 72.7% of individuals with diabetes reported knowledge about complications. A study by Syed et al. [24] reported that 47.3% of the respondents were aware of kidney and eye complications, 23.6% of foot complications, and 17.5% of heart complications. In this study, individuals with complications exhibited significantly higher levels of knowledge than those without complications. The presence of complications in individuals with T2DM leads to significantly higher levels of knowledge about diabetes and its associated risk factors compared to individuals without complications. This relationship suggests that experiencing complications motivates individuals to seek information and education regarding their condition, thereby enhancing their understanding of the disease and its potential consequences. It is also improving their behavior about medication adherence and lifestyle choices. The presence of inertia and ambivalence toward risks is a serious concern. Research supports the notion that many individuals often delay engagement in regular health-promoting activities until faced with significant health events or crises. Theoretical models of health motivation indicate that motivation is a critical precursor to initiating and maintaining health behaviors. However, these models often overlook strategies for engaging those who are unmotivated until they encounter serious health issues, further underscoring the tendency for individuals to wait for a crisis before taking action [25,26].

The limitations of the study include the inherent reporting bias of a self-administered questionnaire, which cannot be entirely eliminated and may lead to instances of overestimating compliance. As the study is cross-sectional, it does not track changes in knowledge or behavior over time. Additionally, various confounding factors, such as economic status, educational level, and the use of alternative systems of medicine, may be present but have not been accounted for in this study. Open-ended questions often depend on the respondent’s verbal ability and memory recall, whereas the respondent can guess specific closed questions. Nevertheless, for extensive population-based studies like this, employing a questionnaire remains the most practical and viable method for collecting such data. Additionally, the participants in the study were sourced exclusively from specialist settings, excluding general practitioners and other healthcare providers who treat individuals with diabetes. This makes the findings more skewed toward people with diabetes with relatively better access and socioeconomic circumstances.

## Conclusions

This study holds significant practical implications for public health interventions in India. The findings from this study and the existing data clearly suggest that we are lagging in our goal for good glycemic control at the population level. There is a clear necessity to expand the workforce of diabetes educators nationwide to address the disease more effectively at a foundational level. We must think of new methodologies of engaging and motivating for educational initiatives, patient counseling, monitoring, and adherence. Health inertia is very real, and among people with T2DM, this invariably leads to very adverse outcomes. As the next steps, we recommend creating a multidisciplinary task force to design and test patient intervention programs and test their effectiveness in a real-world setting.

## Appendices

**Table 5:** Questionnaire and scoring system weightage.

Weightage	Question number	About diabetes mellitus
1	Diabetes is a condition in which the body contains		
	1	A higher level of sugar in the blood than normal	
	-	A lower level of sugar in the blood than normal	
	-	Either a higher or a lower level of sugar in the blood than normal	
	-	I don’t know	
1	The major cause of diabetes is		
	-	An increased availability of insulin in the body	
	2	A decreased availability of insulin in the body	
	-	I don’t know	
-	The symptom(s) of diabetes is/are		
1	3	Increased frequency of urination	
1	4	Increased thirst and hunger	
1	5	Increased tiredness	
1	6	Slow healing of wounds	
-	-	I don’t know	
-	The lifestyle modification(s) required for individuals with diabetes		
1	7	Weight reduction	
1	8	Balanced diet	
1	9	At least 30 minutes of physical exercise daily	
1	10	Stop smoking	
1	11	Stop alcohol intake	
-	-	I don’t know	
1	Upon control of diabetes, the medicines		
	-	Can be stopped immediately	
	-	Can be stopped after a month	
	12	Should continue with medications	
	-	Not sure	
-	Which according to you are complications due to uncontrolled diabetes?		
1	13	Eye problems/Blindness	
1	14	Amputation	
1	15	Kidney disease	
1	16	Heart disease	
1	17	Neuropathy	
1	18	Foot ulcers	
1	19	Shortness of breath	
1	20	Chest pain while walking	
1	21	Sexual problems	
-	-	I don’t know	
-	In an individual with diabetes, high blood pressure can increase or worsen		
1	22	The risk of heart attack	
1	23	The risk of stroke	
1	24	The risk of eye problems	
1	25	The risk of kidney problems	
-	-	I don’t know	
-	Are you aware of blood sugar levels falling below normal when you take medicine?		
1	26	Yes	
-		No	
